# Supplementary material for: Validation of DBFOLD: An efficient algorithm for computing folding pathways of complex proteins
Source: PLoS Comput Biol. 2020 Nov 16;16(11):e1008323. doi: 10.1371/journal.pcbi.1008323 (PMC7704049; doi:10.1371/journal.pcbi.1008323)
Supplement: S1 Text — (PDF) [file pcbi.1008323.s001.pdf]

S1 Text
Details on conditions for applicability of method, Justification of Arrhenius kinetics

Details on conditions for applicability of method

Let us consider the transition between coarse states S_i, with topological configuration i, and S_j with topological configuration j which differs from i by one substructure σ. This transition may generally correspond to either a folding or an unfolding event depending on whether i has one less or one more substructure than j, respectively. We let $s_i^n \in S_i$ denote microstates belonging to coarse state S_i, all of which have topological configuration i and some of which may contain nonnative contacts. We assume that there exists some subset of *hub* microstates $S_i^h \subseteq S_i$ from which a transition to S_j is possible, whereas for all other microstates $s_i^n \in S_i$, this transition is not possible (S1 Fig). This scenario may arise if the transition from S_i to S_j corresponds to a folding event, and non-hub microstates $s_i^n \notin S_i^h$ contain nonnative contacts which impede the formation of σ. Alternatively, if this transition is an unfolding event, then this scenario would arise if microstates $s_i^n \notin S_i^h$ contain nonnative contacts that are strongly energetically coupled to substructure σ and are unlikely to be observed in its absence. We further assume all transitions between microstates are Markovian and satisfy detailed balance. Moreover, if we assume all microstates within the hub rapidly equilibrate with each other, than the hub can also be treated as a microstate, and transitions involving it are Markovian. Under these conditions, all states satisfy the master equation. That is, letting $\lambda^{a,b}$ denote the transition rate between microstates indexed a and b (one or both of which may be a hub state) and letting $\delta^1(s_a)$ contain the indices corresponding to all states that are adjacent to state s_a, we have

$$\frac{d}{dt}P_t(s_a) = \sum_{b\in\delta^1(s_a)} \lambda^{b\to a}P_t(s_b) - \sum_{b\in\delta^1(s_a)} \lambda^{a\to b}P_t(s_a) \tag{1}$$

In case states a and b index the hubs of coarse states S_i and S_j, respectively, then the transition rate between them is denoted $\lambda^{a,b} = \lambda^h_{i\to j}$. In case a indices the hub of state S_i whereas b indices a non-hub state $s_i^b \in S_i$, then we denote $\lambda^{a,b} = \lambda_i^{h\to b}$ and the reverse rate is denoted $\lambda_i^{b\to h}$. Finally, in case both a and b index non-hub states within S_i, then we denote $\lambda^{a,b} = \lambda_i^{a\to b}$.

We wish to compute the mean-first passage time (MFPT), $< \tau_{i\to j} >$ for the transitions from S_i to S_j, and vice versa. In general, owing to high barriers in the free energy landscape, we may only be able to compute this MFPT in one direction via simulation (e.g. unfolding rates can be extrapolated from high temperatures, as discussed in the next section). But given the equilibrium probabilities for the two coarse states $P_{eq}(S_i)$ and $P_{eq}(S_j)$, we can, under certain conditions compute the MFPT for the reverse transition using detailed balance. Two such conditions, which are briefly summarized in the main text, are investigated below in detail:

Condition I

Let us assume that all microstates $s_i^n \in S_i$ rapidly equilibrate with one another relative to the fastest rate of transition to any other topological configurations. Under this separation of timescales, then a given coarse state S_i, will exhibit Markovian behavior. That is, the probability $P_t(S_i)$ of occupying coarse state S_i approxiamtely satisfies the master equation with an effective single-exponential transition rate $k_{i\to j}$ from S_i to S_j is given by:

$$k_{i\to j} = \frac{P_{eq}(S_i^h)}{\sum\limits_{n \text{ s.t. } s_i^n \in S_i} P_{eq}(s_i^n)} \lambda_{i\to j}^h = \frac{P_{eq}(S_i^h)}{P_{eq}(S_i)} \lambda_{i\to j}^h \tag{2}$$

Where $P_{eq}(s)$ refers to the probability of occupying some state s at Boltzmann equilibrium. Likewise, under this limit of fast internal equilibration, the mean-first passage time to reach S_j starting from any microstate within S_i is simply the inverse of the transition rate:

$$< \tau >_{i\to j} = \frac{\sum\limits_{n \text{ s.t. } s_i^n \in S_i} P_{eq}(s_i^n)}{\lambda_{i\to j}^h P_{eq}(S_i^h)} = \frac{P_{eq}(S_i)}{\lambda_{i\to j}^h P_{eq}(S_i^h)} \tag{3}$$

as is the case for any Markovian transition. If so, then assuming both the forward and reverse transitions from S_i to S_j satisfy equation (3), then we find that:

$$\frac{< \tau >_{j\to i}}{< \tau >_{j\to i}} = \frac{P_{eq}(S_j)}{\lambda_{j\to i}^h P_{eq}(S_j^h)} \frac{\lambda_{i\to j}^h P_{eq}(S_i^h)}{P_{eq}(S_i)} = \frac{P_{eq}(S_j)}{P_{eq}(S_i)} \tag{4}$$

To show that equation (2) holds in the limit of fast equilibration, we note that according to the master equation (equation (1)), the time derivative of the probability of occupying state S_i can be expressed as:

$$\frac{d}{dt} P_t(S_i) = -P_t(S_i^h)\lambda_{i\to j}^h + ... = -P_t(S_i) \left(\frac{P_t(S_i^h)}{P_t(S_i)} \right) \lambda_{i\to j}^h + ... \tag{5}$$

Where the first term, which is shown explicitly, gives the probability flux leaving coarse state S_i into some other arbitrary coarse state S_j, while other terms

which are not explicitly shown give the flux leaving S_i into other states, as well as the flux entering from other states. We note that in the limit of fast internal equilibration, terms involving $\lambda^h_{i\to j}$ represent a small perturbation to the master equation. In the unperturbed limit, one of the eigenvectors with zero eigenvalue, denoted $\mathbf{v_0}$ gives the equilibrium probabilities of occupying states within S_i. When the perturbation is turned on, the smallest eigenvalue λ_0 will now have a nonzero value that is nevertheless much smaller than the second smallest eigenvalue λ_1. This results in a significant separation of timescales such that for times $t >> 1/\lambda_1$, the system will show approximate single exponential behavior in which the corresponding perturbed eigenvector, $\tilde{\mathbf{v_0}}$ decays with rate constant λ_0. As such, the ratio $P_t(S^h_i)/P_t(S_i)$ in equation (5) will be given by the (time-independent) ratio of the element in $\tilde{\mathbf{v_0}}$ corresponding to S^h_i, divided by the sum of all elements corresponding to states $s^n_i \in S_i$. For small perturbations, we expect this ratio to be very similar to what it was in $\mathbf{v_0}$, which thus implies that $P_t(S^h_i)/P_t(S_i) \approx P_{eq}(S^h_i)/P_{eq}(S_i)$, in accordance with equation (2).

Condition II

We now relax the condition that microstates within S_i rapidly equilibrate relative to the timescale of transitions to any other coarse state S_j. In this case, we can no longer speak of a single exponential rate for the S_i to S_j transition, as the transition may exhibit multi-exponential kinetics. However, the mean-first passage time to reach S_j nevertheless s provides a useful characteristic timescale for the transition. It turns out that so long as, upon entering S_i, the system always starts in the hub state S^h_i (condition II), then this MFPT will satisfy equation (3), even if the transition to S_j shows multi-exponential kinetics. Thus, if the MFPT is only known for the transition in one direction, then equation (4) can be used to solve for the MFPT in the reverse direction.

To prove that equation (3) holds in this case, we consider a modified version of the master equation (equation (1)) in which we treat S_j as an absorbing boundary and as the only other coarse-state to which S_i can transition. This master equation can be condensed in matrix form, $\dot{\mathbf{P}}_\mathbf{t} = \mathbf{M}\mathbf{P}_\mathbf{t}$, where $\mathbf{P}_\mathbf{t}$ is a vector of state occupancy probabilities as a function of time, $\dot{\mathbf{P}}_\mathbf{t}$ is its time derivative, and $\mathbf{M}$ is the transition matrix whose element $M_{a,b}$ gives the rate of transition from states b to a. Let $\mathbf{M}^T$ denote the transpose of the transition matrix. Then without loss of generality, if we assume the first row of $\mathbf{M}^T$ gives the transition rates from the hub to all states that are adjacent to it, we have

$$M^T_{1,n} = \begin{cases} \lambda_i^{h\to n} & n \in \delta^1(S^h_i) \\ -\lambda^h_{i\to j} - \sum\limits_{m\in\delta^1(S^h_i)} \lambda_i^{h\to m} & n = 1 \\ 0 & \text{otherwise} \end{cases} \tag{6}$$

Meanwhile, the lth element of the nth row will be given by

$$M^T_{n,l} = \begin{cases} -\sum\limits_{m\in\delta^1(S_i^n)} \lambda_i^{n\to m} & l = n \\ \lambda_i^{n\to l} & l \in \delta^1(s_i^n) \\ 0 & \text{otherwise} \end{cases} \tag{7}$$

We want to show that the mean first passage time to coarse state S_j, given that the system starts in the s_i^n, is given by equation (3). To this end, we define a vector τ whose z^{th} element gives the MFPT to the absorbing state S_j assuming the system starts at the zth state at t=0 . This vector satisfies

$$\mathbf{M^T}\tau = -\mathbf{1} \tag{8}$$

Where $\mathbf{M^T}$ refers to the tranpose of $\mathbf{M}$ and $-\mathbf{1}$ is a vector of negative ones with the same dimension as τ. We are interested in solving for the first element of this vector, assuming an arbitrary topology for the microstates $s_i^n \in S_i$. This can be accomplished by augmenting the matrix $\mathbf{M^T}$ with a matrix $\mathbf{A}$, which starts off as the identity, and row-reducing using Gauss-Jordan elimination to obtain the inverse $(\mathbf{M^T})^{-1}$.

We row-reduce via an iterative process as follows: During each step of row reduction, we consider the set $\delta^k(S_i^h)$, which contains the indices for all states that are removed from state S_i^h via k degrees of separation. Every row whose index n is an element of $\delta^k(S_i^h)$ is scaled by a factor $P_{eq}(s_i^n)/P_{eq}(S_i^h)$, then added to the first row. We let $\mathbf{M^{T,k}}$ denote the matrix $\mathbf{M^T}$ as it stands after k such iterations of row reduction, and $\mathbf{A^k}$ denote the same for the augmentation (noting that $\mathbf{A^0}$ is the identity matrix). Then clearly, after k rounds of row-reduction, we have

$$A^k_{1,n} = \begin{cases} \frac{P_{eq}(s_i^n)}{P_{eq}(S_i^h)} & n \in \delta^1(S_i^h) \cup \delta^2(S_i^h) \cup ... \cup \delta^k(S_i^h) \\ 0 & n \notin \delta^1(S_i^h) \cup \delta^2(S_i^h) \cup ... \cup \delta^k(S_i^h) \end{cases} \tag{9}$$

And we claim that

$$M^{T,k}_{1,n} = \begin{cases} -\frac{P_{eq}(s_i^n)}{P_{eq}(S_i^h)} \sum\limits_{m\in\delta^{k+1}(S_i^h)} \lambda_i^{n\to m} & n \in \delta^k(S_i^h) \\ \sum\limits_{m\in\delta^k(S_i^h)} \frac{P_{eq}(s_i^m)}{P_{eq}(S_i^h)} \lambda^{m\to n} & n \in \delta^{k+1}(S_i^h) \\ -\lambda^h_{i\to j} & n = 1 \\ 0 & \text{otherwise} \end{cases} \tag{10}$$

If so, then we see that once we reach k such that $\delta^{k+1}(S_i^h)$ is empty, then we can divide the first row of both $\mathbf{M^{T,k}}$ and $\mathbf{A_{1,n}}$ by $-\lambda^h_{i\to j}$, at which point the

first row of $M_{1,n}^{T,k}$ is fully reduced. We thus find that the first row of $(\mathbf{M^T})^{-1}$ is the identity multiplied by $-P_{eq}(S_i)/(\lambda_{i\to j}^h P_{eq}(S_i^h))$. To solve equation (8), we simply need to multiply this first row by -1, at which point we obtain equation (3), completing the proof.

We prove equation (10) by induction.

Base Case $k = 1$: From equations (6) and (7), we note that after the first round of row reduction, we have

$$M_{1,n}^{T,1} = \begin{cases} \lambda_i^{h\to n} + \sum\limits_{m\in\delta^1(S_i^h)} \frac{P_{eq}(s_i^m)}{P_{eq}(S_i^h)} \lambda_i^{m\to n} - \frac{P_{eq}(s_i^n)}{P_{eq}(S_i^h)} \sum\limits_{m\in\delta^1(s_i^n)} \lambda_i^{n\to m} & n \in \delta^1(S_i^h) \\ \sum\limits_{m\in\delta^1(S_i^h)} \frac{P_{eq}(s_i^m)}{P_{eq}(S_i^h)} \lambda^{m\to n} & n \in \delta^2(S_i^h) \\ -\lambda_{i\to j}^h - \sum\limits_{m\in\delta^1(S_i^h)} \lambda_i^{h\to m} + \sum\limits_{m\in\delta^1(S_i^h)} \frac{P_{eq}(s_i^m)}{P_{eq}(S_i^h)} \lambda_i^{m\to h} & n = 1 \\ 0 & \text{otherwise} \end{cases} \tag{11}$$

The second case above clearly satisfies equation (10). In the third case, we note that by detailed balance, $\lambda_i^{m\to h} \frac{P_{eq}(s_i^m)}{P_{eq}(S_i^h)} = \lambda_i^{h\to m}$. Thus, the second and third sums cancel, proving equation (10) for this case. Finally, to show that the first case above satisfies the first case in equation (10), we rewrite it as follows

$$\begin{aligned} &\lambda_i^{h\to n} + \sum_{m\in\delta^1(S_i^h)} \frac{P_{eq}(s_i^m)}{P_{eq}(S_i^h)} \lambda_i^{m\to n} - \frac{P_{eq}(s_i^n)}{P_{eq}(S_i^h)} \sum_{m\in\delta^1(S_i^n)} \lambda_i^{n\to m} = \\ &\lambda_i^{h\to n} + \sum_{m\in\delta^1(S_i^h)} \frac{P_{eq}(s_i^m)}{P_{eq}(S_i^h)} \lambda_i^{m\to n} - \lambda_i^{h\to n} \\ &\quad - \sum_{m\in\delta^1(S_i^h)} \frac{P_{eq}(s_i^m)}{P_{eq}(S_i^h)} \lambda_i^{m\to n} - \frac{P_{eq}(s_i^n)}{P_{eq}(S_i^h)} \sum_{m\in\delta^2(S_i^h)} \lambda_i^{n\to m} \\ = &-\frac{P_{eq}(s_i^n)}{P_{eq}(S_i^h)} \sum_{m\in\delta^2(S_i^h)} \lambda_i^{n\to m} \end{aligned}$$

Where in the first equality we have split the third sum from the LHS and used detailed balance to re-express terms in the resulting second sum. This proves the base case.

Inductive step We now consider the kth round of row reduction. By our

inductive hypothesis, prior to this round we have

$$M_{1,n}^{T,k-1} = \begin{cases} -\frac{P_{eq}(s_i^n)}{P_{eq}(S_i^h)} \sum_{m\in\delta^k(S_i^h)} \lambda_i^{n\to m} & n \in \delta^{k-1}(S_i^h) \\ \\ \sum_{m\in\delta^{k-1}(S_i^h)} \frac{P_{eq}(s_i^m)}{P_{eq}(S_i^h)} \lambda^{m\to n} & n \in \delta^k(S_i^h) \\ \\ -\lambda_{i\to j}^h & n = 1 \\ \\ 0 & \text{otherwise} \end{cases} \tag{12}$$

Then from the above and equation (7), we see that after the row-reduction, we have

$$M_{1,n}^{T,k} = \begin{cases} -\frac{P_{eq}(s_i^n)}{P_{eq}(S_i^h)} \sum_{m\in\delta^k(S_i^h)} \lambda_i^{n\to m} + \sum_{m\in\delta^k(S_i^h)} \frac{P_{eq}(s_i^m)}{P_{eq}(S_i^h)} \lambda_i^{m\to n} & n \in \delta^{k-1}(S_i^h) \\ \\ \sum_{m\in\delta^{k-1}(S_i^h)} \frac{P_{eq}(s_i^m)}{P_{eq}(S_i^h)} \lambda^{m\to n} + \sum_{m\in\delta^k(S_i^h)} \frac{P_{eq}(s_i^m)}{P_{eq}(S_i^h)} \lambda_i^{m\to n} - \frac{P_{eq}(s_i^n)}{P_{eq}(S_i^h)} \sum_{m\in\delta^1(S_i^n)} \lambda_i^{n\to m} & n \in \delta^k(S_i^h) \\ \\ \sum_{m\in\delta^k(S_i^h)} \frac{P_{eq}(s_i^m)}{P_{eq}(S_i^h)} \lambda_i^{m\to n} & n \in \delta^{k+1}(S_i^h) \\ \\ -\lambda_{i\to j}^h & n = 1 \\ \\ 0 & \text{otherwise} \end{cases} \tag{13}$$

The third and fourth cases clearly satisfy eq. (10) Let us now consider the first case where $n \in \delta^{k-1}(S_i^h)$. If we multiply all terms in the second sum by $P_{eq}(s_i^n)/P_{eq}(s_i^n)$ and note that by detailed balance, $P_{eq}(s_i^m)/P_{eq}(s_i^n)\lambda_i^{m\to n} = \lambda_i^{n\to m}$, then we find that the second sum cancels the first. Thus we have proven the "otherwise" case in equation (10). The final case that remains to be

proven is for $n \in \delta^k(S_i^h)$. We note that this case can be expressed as follows

$$\sum_{m\in\delta^{k-1}(S_i^h)} \frac{P_{eq}(s_i^m)}{P_{eq}(S_i^h)}\lambda^{m\to n} + \sum_{m\in\delta^{k}(S_i^h)} \frac{P_{eq}(s_i^m)}{P_{eq}(S_i^h)}\lambda_i^{m\to n} - \frac{P_{eq}(s_i^n)}{P_{eq}(S_i^h)} \sum_{m\in\delta^{1}(S_i^n)} \lambda_i^{n\to m}$$

$$= \sum_{m\in\delta^{k-1}(S_i^h)} \frac{P_{eq}(s_i^m)}{P_{eq}(S_i^h)}\lambda^{m\to n} + \sum_{m\in\delta^{k}(S_i^h)} \frac{P_{eq}(s_i^m)}{P_{eq}(S_i^h)}\lambda_i^{m\to n} - \sum_{m\in\delta^{k-1}(S_i^h)} \frac{P_{eq}(s_i^m)}{P_{eq}(S_i^h)}\lambda_i^{m\to n}$$

$$- \sum_{m\in\delta^{k}(S_i^h)} \frac{P_{eq}(s_i^m)}{P_{eq}(S_i^h)}\lambda_i^{m\to n} - \frac{P_{eq}(s_i^n)}{P_{eq}(S_i^h)} \sum_{m\in\delta^{k+1}(S_i^h)} \lambda_i^{n\to m}$$

$$= -\frac{P_{eq}(s_i^n)}{P_{eq}(S_i^h)} \sum_{m\in\delta^{k+1}(S_i^h)} \lambda_i^{n\to m}$$

Where in the first equality we have split the third sum from the LHS and again multiplied all terms except those in the fourth sum by $P_{eq}(s_i^n)/P_{eq}(s_i^n)$ and used detailed balance, resulting in the cancellation of all sums except the last. This completes the proof.

Justification of Arrhenius kinetics

We now consider an unfolding transition between coarse states S_i and S_j in which substructure σ is disrupted. As above, we assume that this transition can only occur between the respective hub states $S_i^h \subseteq S_i$ and $S_j^h \subseteq S_j$ and that these hub states rapidly equilibrate internally. We further assume that the transition rate between these hubs $\lambda_{i\to j}^h(T)$ (with the temperature-dependence now explicitly shown) is given by the Arrhenius equation:

$$\log \lambda_{i\to j}^h(T) = \log \lambda_{i\to j}^{h,0} - \frac{E_\sigma^*}{k_B T} \tag{14}$$

Where $\lambda_{i\to j}^{h,0}$ is a temperature-independent prefactor, E_σ^* is the activation energy for this transition, which is expected to be associated with the breaking of substructure σ, and $k_B T$ is the Boltzmann constant multiplied by the absolute temperature as before. Now, let us assume that in addition to this hub, S_i also contains non-hub microstates s_i^n from which transitions to S_j cannot occur (or occur very slowly compared to the transition via the hub). Does the overall (inverse) mean-first passage time to escape S_i still satisfy the Arrhenius equation, despite the presence of these off-pathway microstates? Let us assume that either Condition I or II holds for this transition, such that mean-first passage time is given by equation (3). Furthermore, we make the simplifying assumption that the ratio of the equilibrium probabilities of states s_i^n and S_i^h has a simple Van't-Hof like temperature dependence

$$\frac{P_{eq}(s_i^n)}{P_{eq}(S_i^h)}(T) = \exp\left(\frac{E_n}{k_B T} - S_n\right) \tag{15}$$

Where E_n is the energy difference (assumed to be temperature-independnet) between S_i^h and s_i^n, and S_n is the entropy difference between this pair of states. Plugging equation (15) into equation (3), inverting and taking the log of both sides, we obtain

$$\log < \tau_{i\to j} >^{-1} (\beta) = \log \lambda_{i\to j}^{h,0} - \beta E_\sigma^* - \log \left[1 + \sum_n k_0^n e^{\beta E_n} \right] \tag{16}$$

Where we have made the replacements $\beta = 1/k_B T$ and $k_0^n = e^{-S_n}$. In general, this predicts a nonlinear relationship between $\log < \tau_{i\to j} >^{-1} (T)$ and temperature, implying the Arrhenius equation cannot be used. However, let us consider the special case where, over the entirety of our temperature range of interest, one specific off-pathway microstate s_i^m has a significantly greater equilibrium occupancy probability than all others, including microstates within the hub. That is, for all $n \neq m$, we have $k_0^n e^{-\beta(E_m - E_n)}/k_o^m << 1$, and likewise $e^{-\beta E_m}/k_o^m << 1$. Differentiating equation (16) with respect to β, we obtain

$$\frac{\partial}{\partial \beta} \log < \tau_{i\to j} >^{-1} (\beta) = -E_\sigma^* - E_m^* - \frac{E_m e^{-\beta E_m}/k_o^m + \sum_{n\neq m}(E_m - E_n) k_0^n e^{-\beta(E_m - E_n)}/k_o^m}{1 + e^{-\beta E_m}/k_o^m + \sum_{n \neq m} k_0^n e^{-\beta(E_m - E_n)}/k_o^m} \tag{17}$$

Noting that all terms in the numerator of the fraction are much smaller than one, we treat this fraction as a negligible correction and thus have

$$\frac{\partial}{\partial \beta} \log < \tau_{i\to j} >^{-1} (\beta) \approx -E_\sigma^* - E_m^* \tag{18}$$

Thus, so long as one off-pathway microstate s_i^n dominates at all temperatures of interest, we recover Arrhenius-like behavior with an effective total activation energy that includes the energy needed to break nonnative contacts (E_m^*) and that needed to break σ (E_σ^*).
