# Supplementary figures and images for: Validation of DBFOLD: An efficient algorithm for computing folding pathways of complex proteins
Source: PLoS Comput Biol. 2020 Nov 16;16(11):e1008323. doi: 10.1371/journal.pcbi.1008323 (PMC7704049; doi:10.1371/journal.pcbi.1008323)

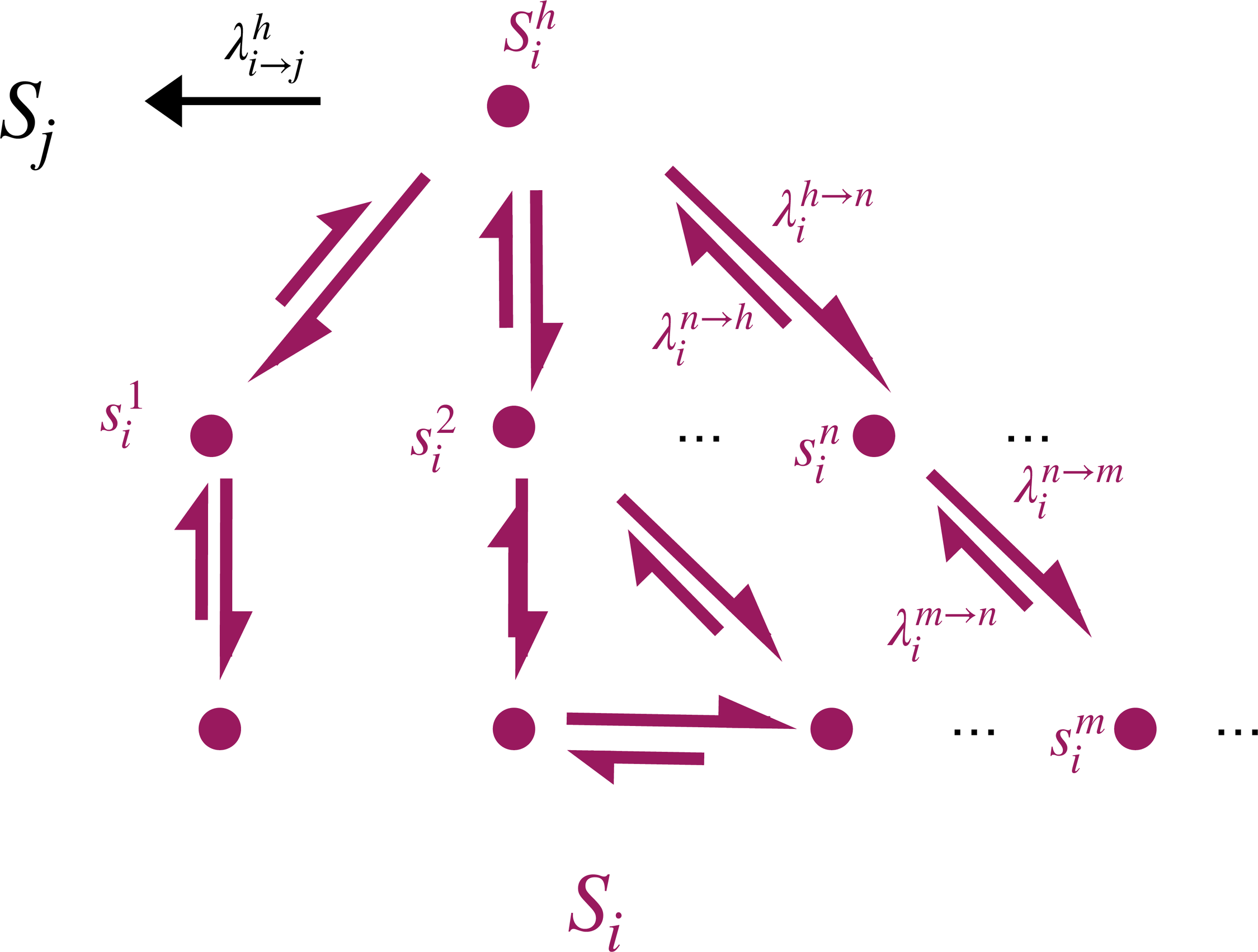

Supplement: S1 Fig — Si and Sj represent coarse states, each of which is composed of the set of all microstates with topological configuration i or j, respectively. Si can transition to Sj if the two differ by the formation/breaking of one substructre. Sih⊆Si represents the subset of microstates within Si from which transitions to states in Sj are possible (i.e. the hub state). All other microstates sin∈Si cannot transition to Sj owing to nonnative contacts that interfere with folding/unfolding of the required native substructure. Transitions between states are indicated by double arrows and rates denoted by the variable λ. For further details, see S1 Text. (TIF) [file pcbi.1008323.s002.tif]

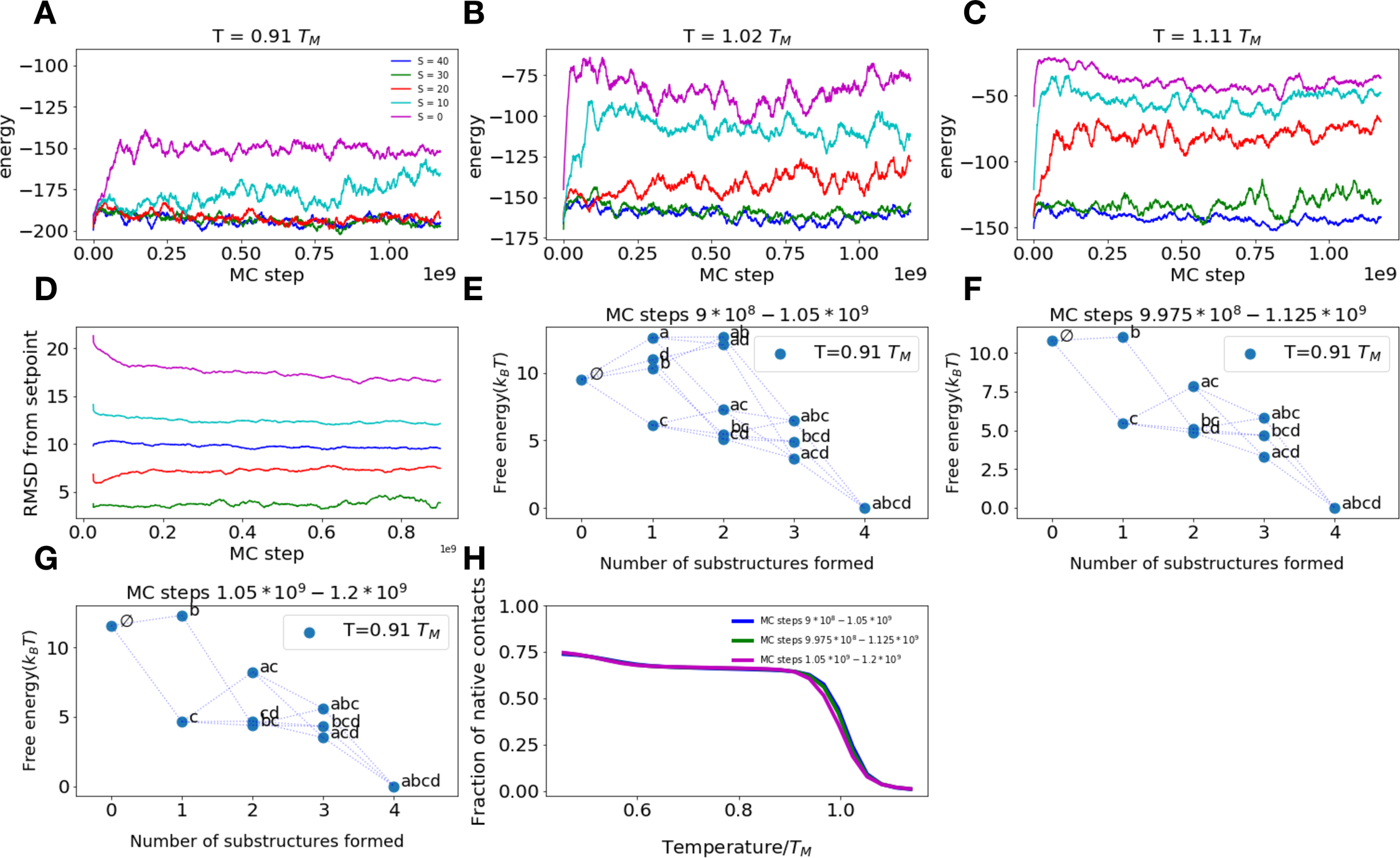

Supplement: S2 Fig — (A)-(C) Energy is plotted as a function of Monte Carlo step, averaged over a sliding window of 50 million Monte Carlo steps, at three temperatures shown above plots, where TM is the melting temperature. At each temperature, trajectories for each umbrella setpoint are shown (see legend in left-most panel). At around 1 billion MC steps, these sliding-window averaged energies cease changing substantially, indicative of convergence. (D) For each setpoint, we compute the root-mean-squared deviation between the number of native contacts and the respective setpoint value averaged over all temperatures and over a sliding window of 50 million MC steps. This quantity is plotted as a function of MC step, with different colors corresponding to setpoints as in panel (A). This RMSD stops varying after about 1 billion MC steps, indicating convergence. (E)-(G) Potentials of mean force (PMF) as a function of topological configuration are plotted at a simulation temperature of T = 0.91 TM (as in main text Fig 3C), but we now vary the window of simulation timesteps that is used to compute the PMFs, namely we use either steps 900 million through 1.05 billion (E), 975 million through 1.125 billion (F), or 1.05 billion through 1.2 billion (G). Quantitative similarity between these PMFs indicates that thermodynamic quantities are well converged. (H) Thermally averaged fraction of native contacts as a function of simulation temperature (as in main text Fig 3B) using the same MC timestep windows as in panels (E) -(G) (see legend). These curves are nearly superimposable, indicating that thermodynamic quantities are well converged. (TIF) [file pcbi.1008323.s003.tif]

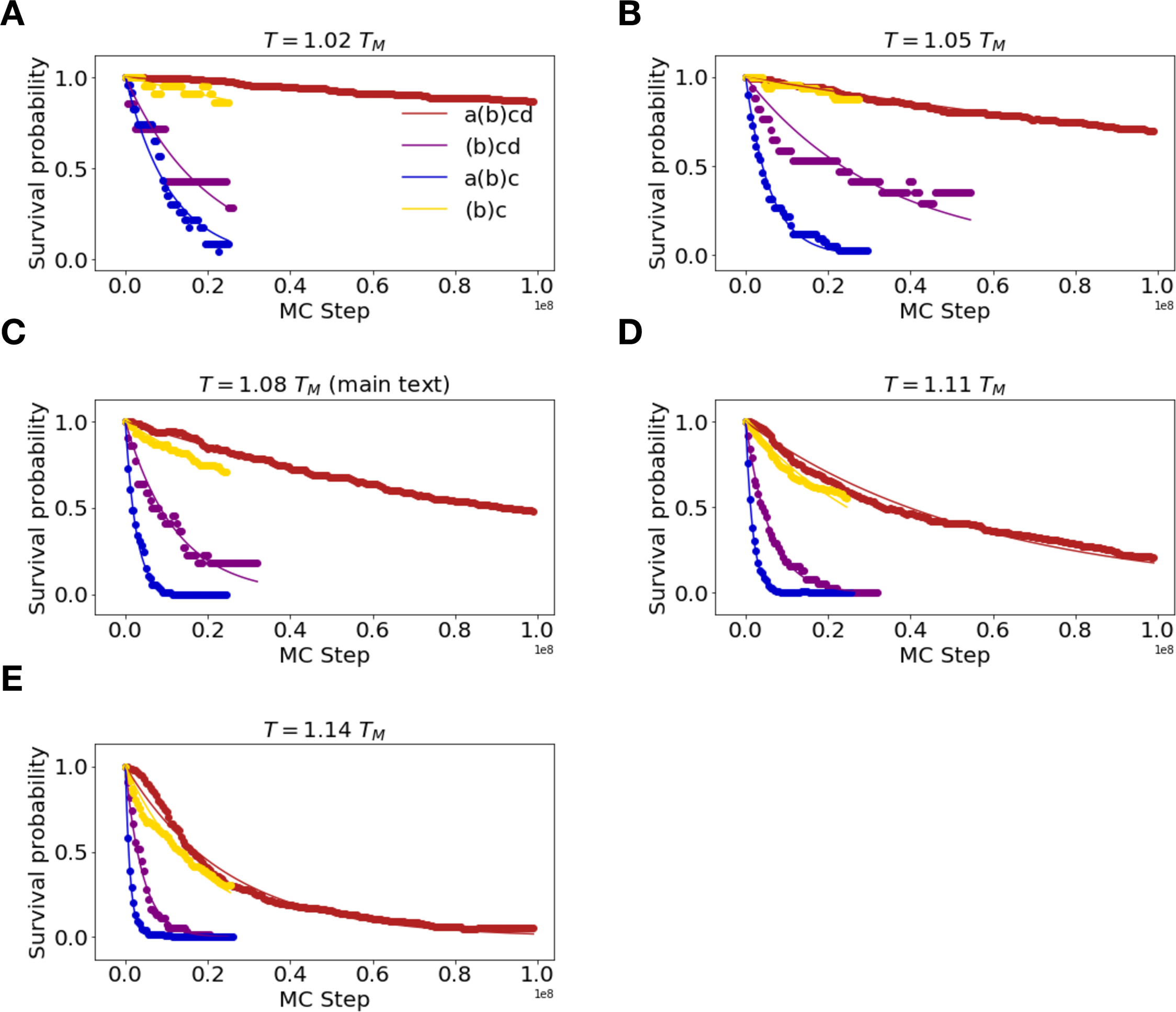

Supplement: S3 Fig — (A)-(E) Survival probability as a function of Monte Carlo time for each cluster (dots) alongside exponential fits (solid lines) during unfolding simulations, as in main text Fig 3C, at simulation temperatures indicated above the respective panels. Panel (C) shows data at a simulation temperature of 1.08 TM, as in the main text. We note that survival curves for a cluster C at temperature T can only be meaningfully computed over a MC time interval τ=τtraj-maxi(τi0), where τtraj = 108 MC steps is the total duration of each trajectory, τi0 is the first MC timestep at which trajectory i (at temperature T) samples cluster C, and thus maxi(τi0) is the latest MC timepoint at which any trajectory at temperature T reaches cluster C for the first time. For all clusters except abcd (at which all trajectories are initialized), τ is shorter than the total simulation trajectory, hence these clusters’ survival curves are truncated. Most curves show good fits to a single-exponential decay. The fits for cluster (b)cd are worse because the (b)cd to (b)c transition satisfies condition II, but not condition I, implying multi-exponential kinetics. We note that these exponential fits are merely used to assess whether clusters satisfy condition I, and not to infer transition rates–these rates are instead inferred using Eqs (10) and (11) in the main text. (TIF) [file pcbi.1008323.s004.tif]

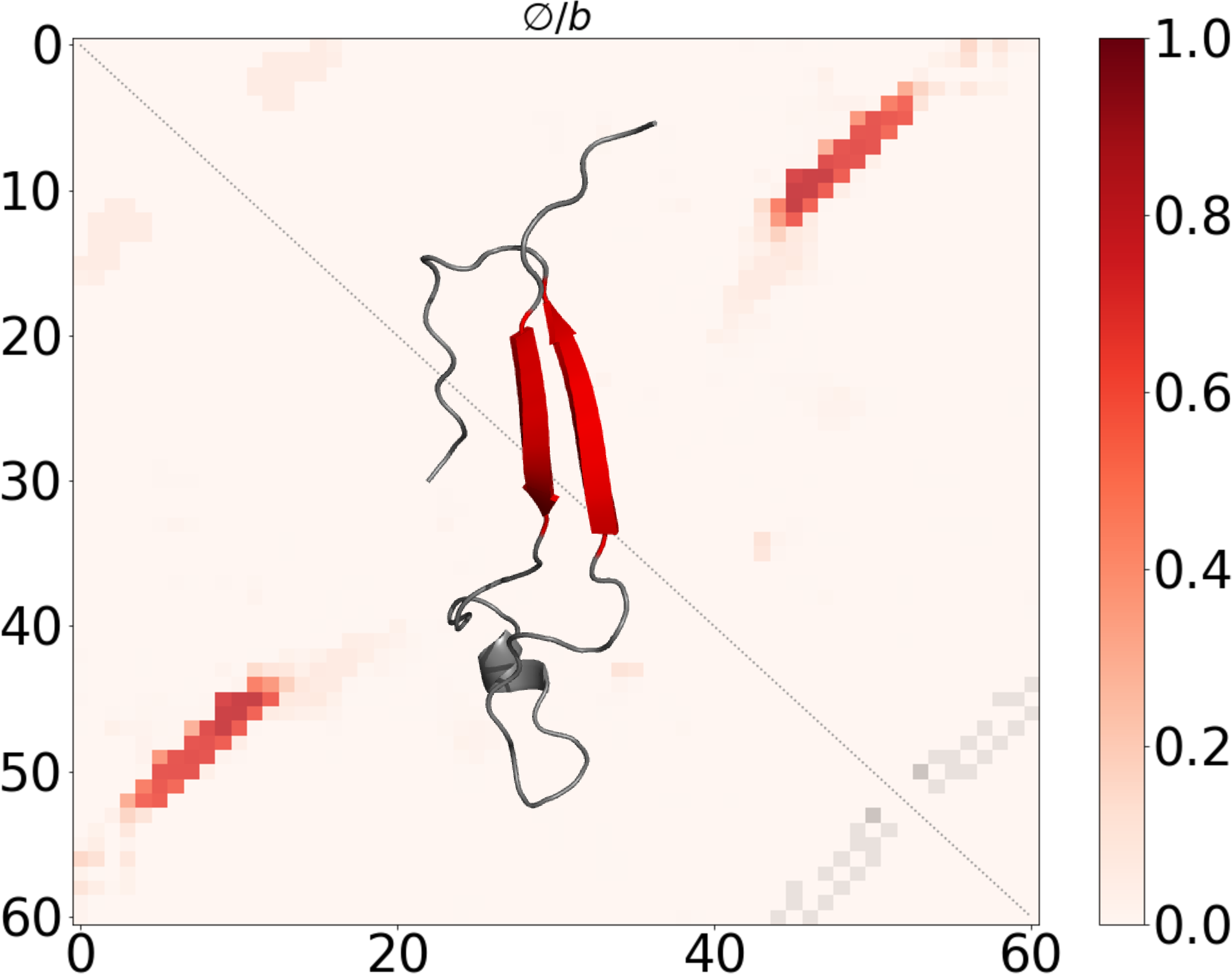

Supplement: S4 Fig — Average nonnative contact map (as in main text Fig 5A) for snapshots assigned to cluster ∅/b at temperatures around T ≈ 0.85 TM, alongside an example of such a snapshot with nonnative contacts highlighted. Residues that participate in substructure c, the first to form in the folding pathway, are outlined in gray. The predominant nonnative contacts do not impede the impede the formation of substructure c–this is further evidenced by the fact that these same nonnative contacts are observed in cluster (b)c (See Fig 4A). (TIF) [file pcbi.1008323.s005.tif]

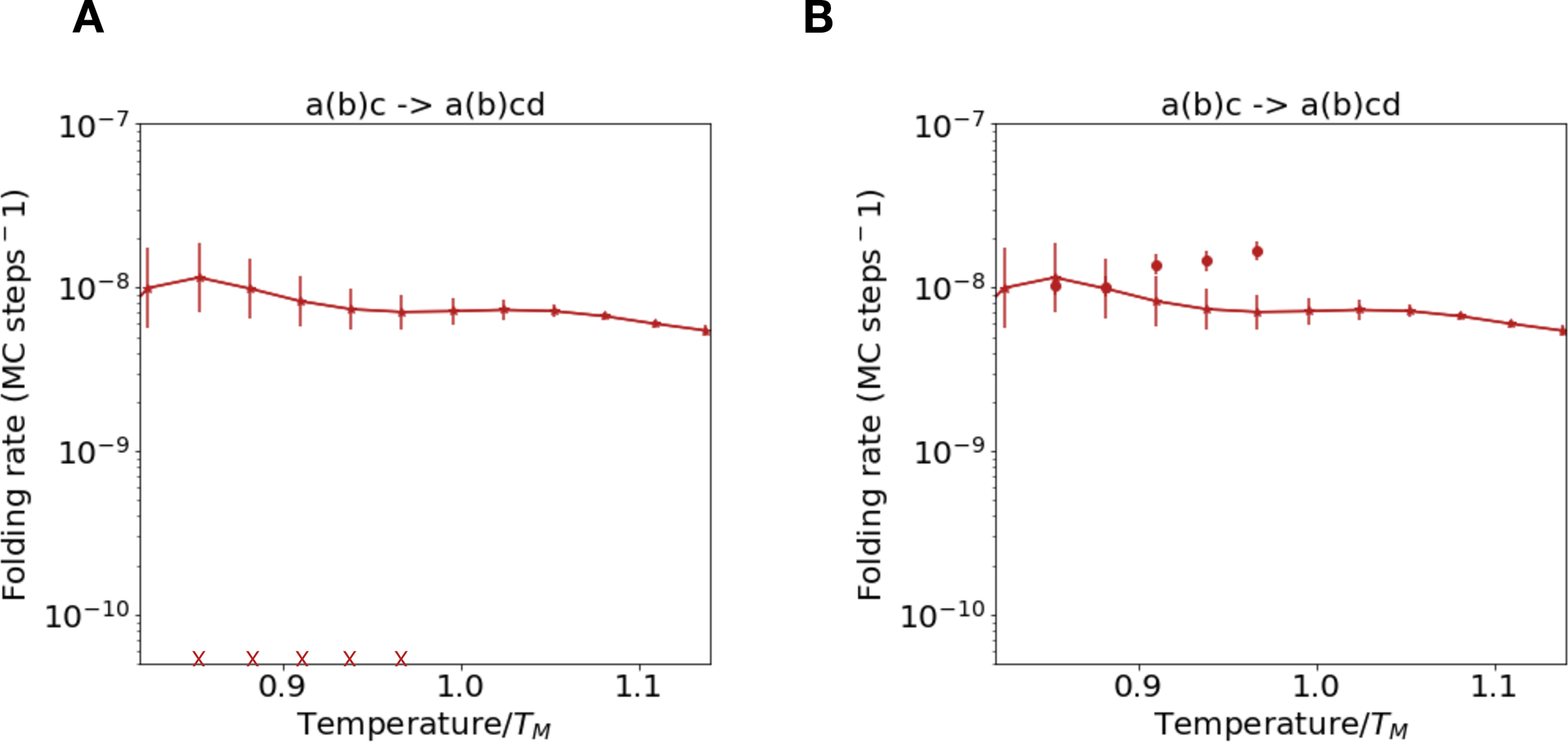

Supplement: S5 Fig — (A) Predicted inverse mean-first passage times (MFPTs) to folding (markers with errorbars connected by lines), alongside observed inverse MFPTs from serial refolding simulations (disconnected round markers), are shown as a function of simulation temperature for the transition from clusters a(b)c to a(b)cd (analogous to main text Fig 6). Although the algorithm predicts inverse folding times of approximately 10−8 MC steps−1 (implying that roughly one folding transition should occur per simulation trajectory) at physiologically-reasonable temperatures, in reality no transitions are observed at all at any temperature (as indicated by X’s near the x-axis), implying that the true folding rate is likely less than 10−10 MC steps−1. This significant discrepancy occurs because, in the context of serial refolding simulations, neither condition I nor II is satisfied for the a(b)c to a(b)cd transition. (B) Same as (A), but markers now show observed a(b)c to a(b)cd inverse mean first passage times within simulations that are initialized from a(b)c snapshots drawn from temperatures above the melting temperature with no more than two nonnative contacts. This setup artificially ensures that Condition II holds for this transition. In contrast to panel (A), all observed transition rates now lie within a factor of two of the predicted rates, indicating significantly improved predictions. (TIF) [file pcbi.1008323.s006.tif]

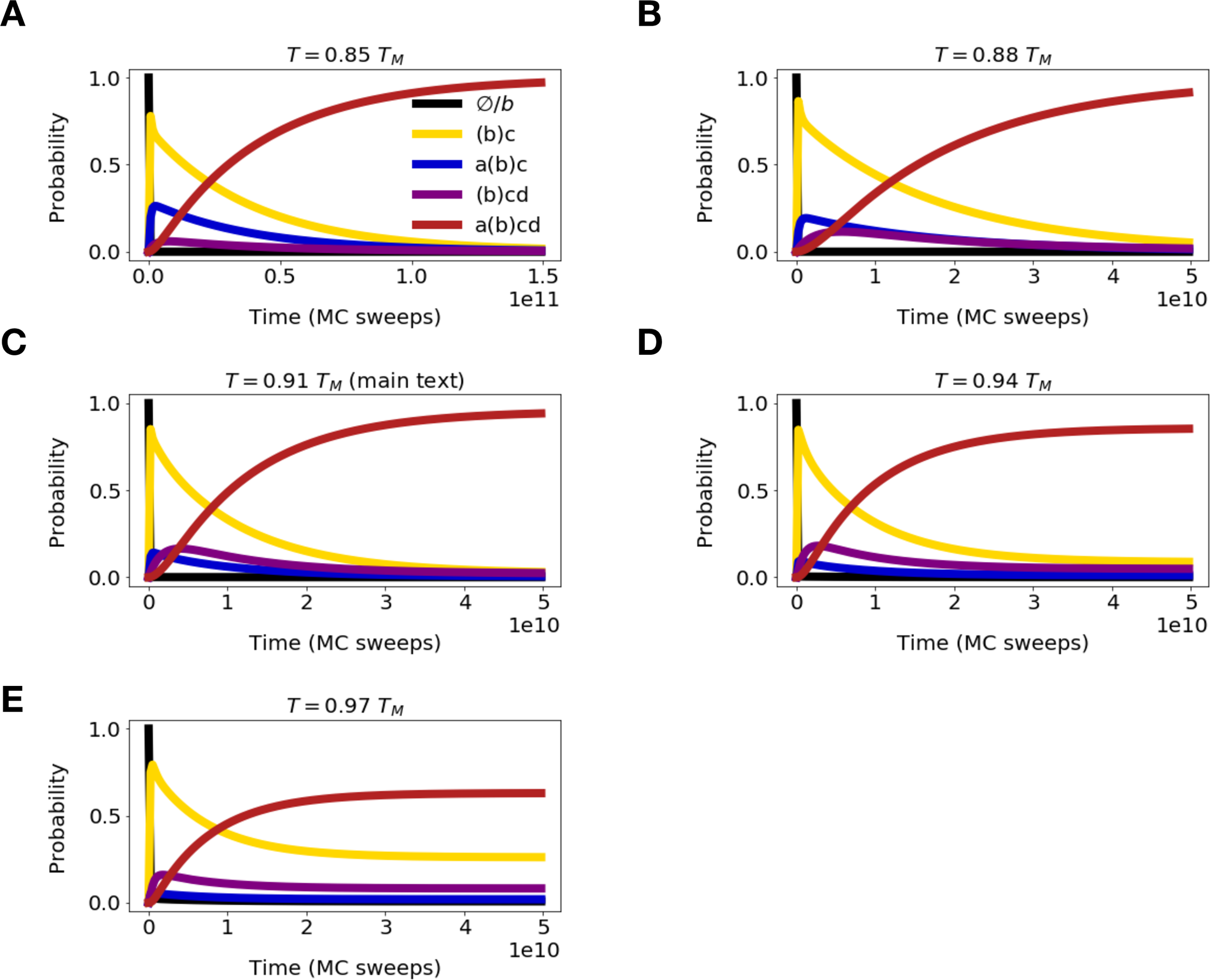

Supplement: S6 Fig — (A)—(E) As in main text Fig 6E, we solve the master equation, which incorporates folding and unfolding rates between clusters computed as described in the main text, for the probability of occupying different clusters as a function of time at five different physiologically reasonable temperatures indicated above the respective panels. Panel (C) shows the solution at T = 0.91 TM as in the main text. All temperatures show qualitatively similar kinetics, although quantitative details slightly differ between temperatures. In particular, at lower temperatures, relaxation dynamics are slower due to increased stability of nonnative contacts (note the different x-scale in panel (A)), but the fully folded state is also more stable, leading to a higher final equilibrium population of cluster a(b)cd. (TIF) [file pcbi.1008323.s007.tif]

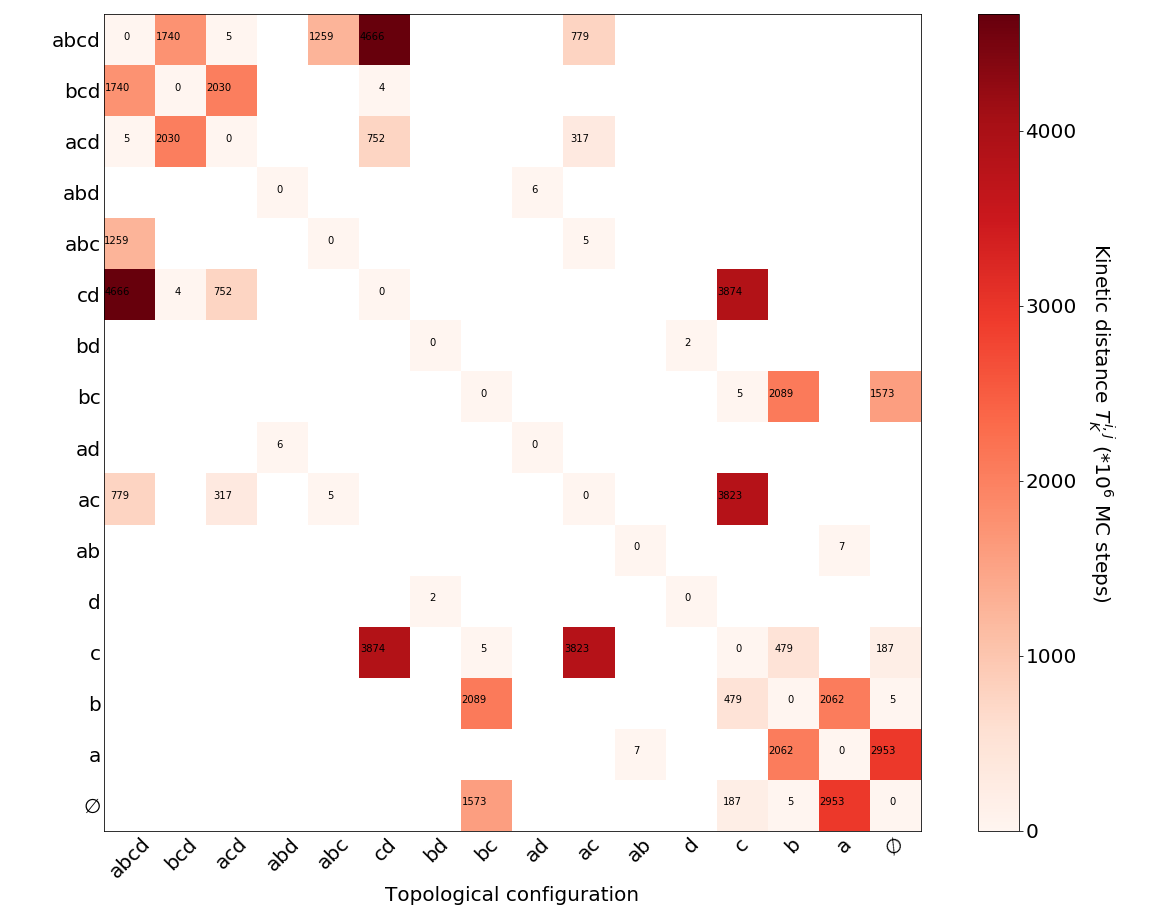

Supplement: S7 Fig — The kinetic distance TKi,j–namely the average time to transition between topological configurations i and j in either direction at any temperature conditioned on the fact that the system is in one of the two states –is shown between every pair of topological configurations observed in unfolding simulations. Values written out inside the heatmap are in units of millions of MC steps. This provides a metric for the speed at which topological configurations exchange with each other. We note a significant separation between the fastest timescales of exchange (less than 10 million MC steps) and all slower timescales (greater than 150 million MC steps). Thus, a kinetic threshold of TA = 100*106 MC steps (see main text Methods section) produces meaningful kinetic clusters. (TIF) [file pcbi.1008323.s008.tif]

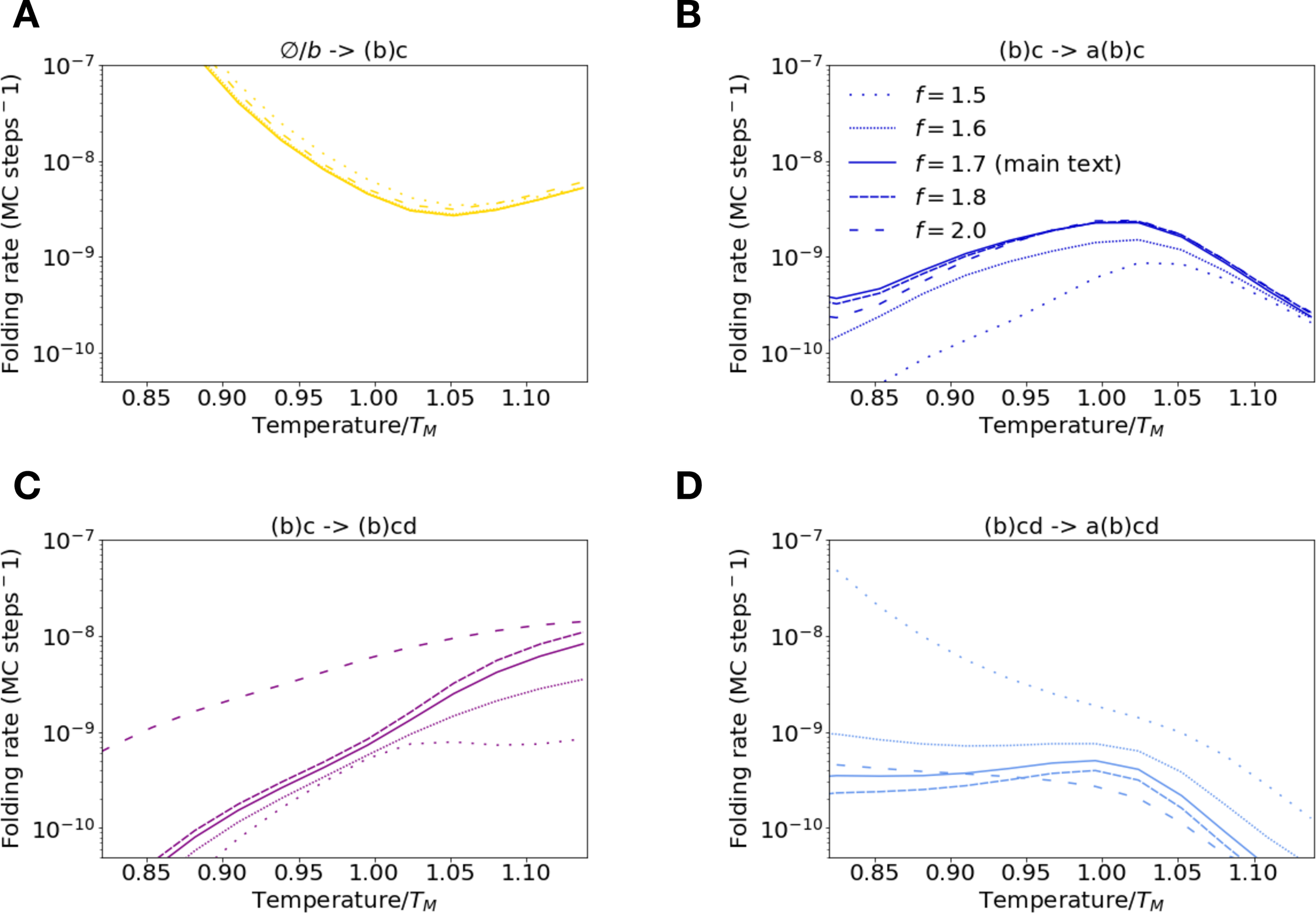

Supplement: S8 Fig — (A)-(D) Predicted folding rate as a function of simulation temperature for each transition that satisfies either condition I or II (as in main text Fig 6) for different values of f, which refers to the maximum allowed ratio of the average distance between residues assigned to a substructure in a snapshot divided by that same average distance in the equilibrated file such that the substructure is deemed folded in that snapshot (See main text Materials and methods, subsection “Substructure analysis”). Different linestyles indicate different values of f (as per legend in panel B), We note that for all transitions, small deviations of f away from the value used in the main text, namely f = 1.7, do not significantly change the predicted folding rates, indicating that f = 1.7 is a reasonable value. However, larger deviations in f produce more significant changes to these predictions. If our threshold for declaring a substructure folded is too strict (f ∼ ≤1.5), then the algorithm tends to overpredict unfolding events, whereas too lenient a threshold (f ∼ ≥2) causes the algorithm to underpredict such events. We note that for the extreme values of f, the kinetic clusters slightly change. Namely when f = 1.5 all clusters except a(b)cd include only the respective topological configuration in which b is unfolded (e.g. (b)cd now only includes cd), as b is rarely declared folded under this strict threshold. Conversely, when f = 2, clusters a(b)cd and (b)cd include only the respective configuration in which b is folded, as b is rarely declared unfolded under this lenient threshold. Finally we note that higher f values may reasonably be used for predominantly helical proteins where interactions between contacting residues typically involve sidechains, as opposed to backbone hydrogen bonds as in predominantly sheet proteins such as protein G. (TIF) [file pcbi.1008323.s009.tif]

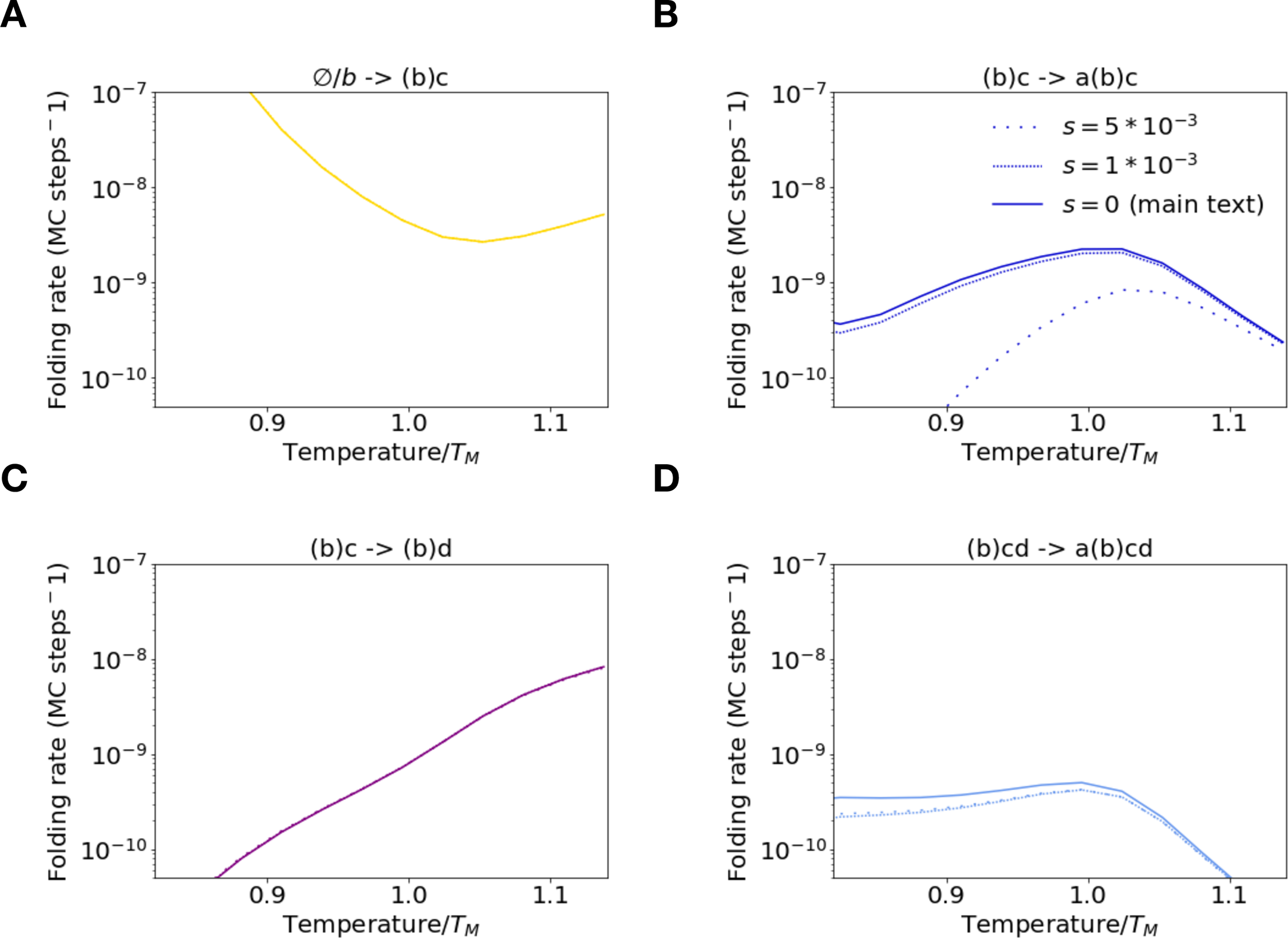

Supplement: S9 Fig — Predicted folding rate as a function of simulation temperature for each transition that satisfies either condition I or II (as in main text Fig 6) for different values of s. Following initial assignment, all topological configurations that encompass less than a fraction s of snapshots over all unfolding simulations are reassigned to the most similar topological configuration that is represented at a fractional prevalence greater than s (see main text Materials and methods, subsection “Computing and extrapolating unfolding rates”). This is useful for larger proteins to reduce the number of unfolding rate parameters. In the main text, a value of s = 0 is used (no reassignment), but these panels show that a value of s = 0.001 does not drastically change any results. This value reassigns states that are not part of the two predominant folding/unfolding pathways, but leave intact assignments for states belonging to these pathways. Meanwhile, a value of s = 0.005 does not drastically change results with the exception of the (b)c → a(b)c transition rates. This is because, for this relatively large value of s, configuration a(b)c, which is observed with frequency less than 0.005 among all snapshots despite belonging to one of the dominant folding/unfolding pathways, gets reassigned. These results therefore indicate that the value of s should always be small enough that topological configurations belonging to the dominant pathways are not reassigned. (TIF) [file pcbi.1008323.s010.tif]

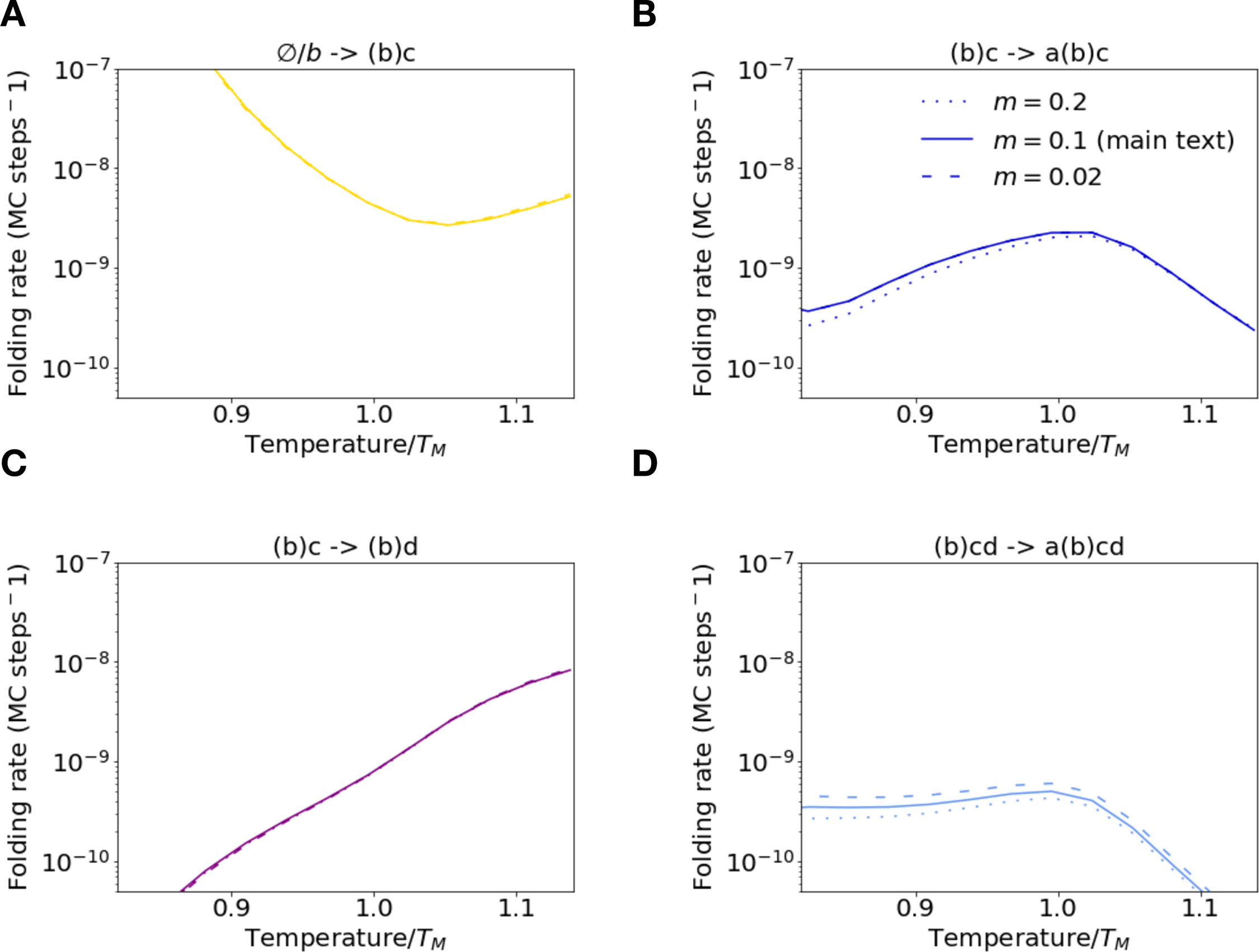

Supplement: S10 Fig — Predicted folding rate as a function of simulation temperature for each transition that satisfies either condition I or II (as in main text Fig 6) for different values of m, the misassignemnt probability in the hidden Markov model used to reduce misassignment of snapshots to incorrect topological configurations (see main text Methods section, subsection Computing and extrapolating unfolding rates). A value of m = 0.1 is used in the main text, but these panels show that our results are robust even if m is varied over an order of magnitude. (TIF) [file pcbi.1008323.s011.tif]

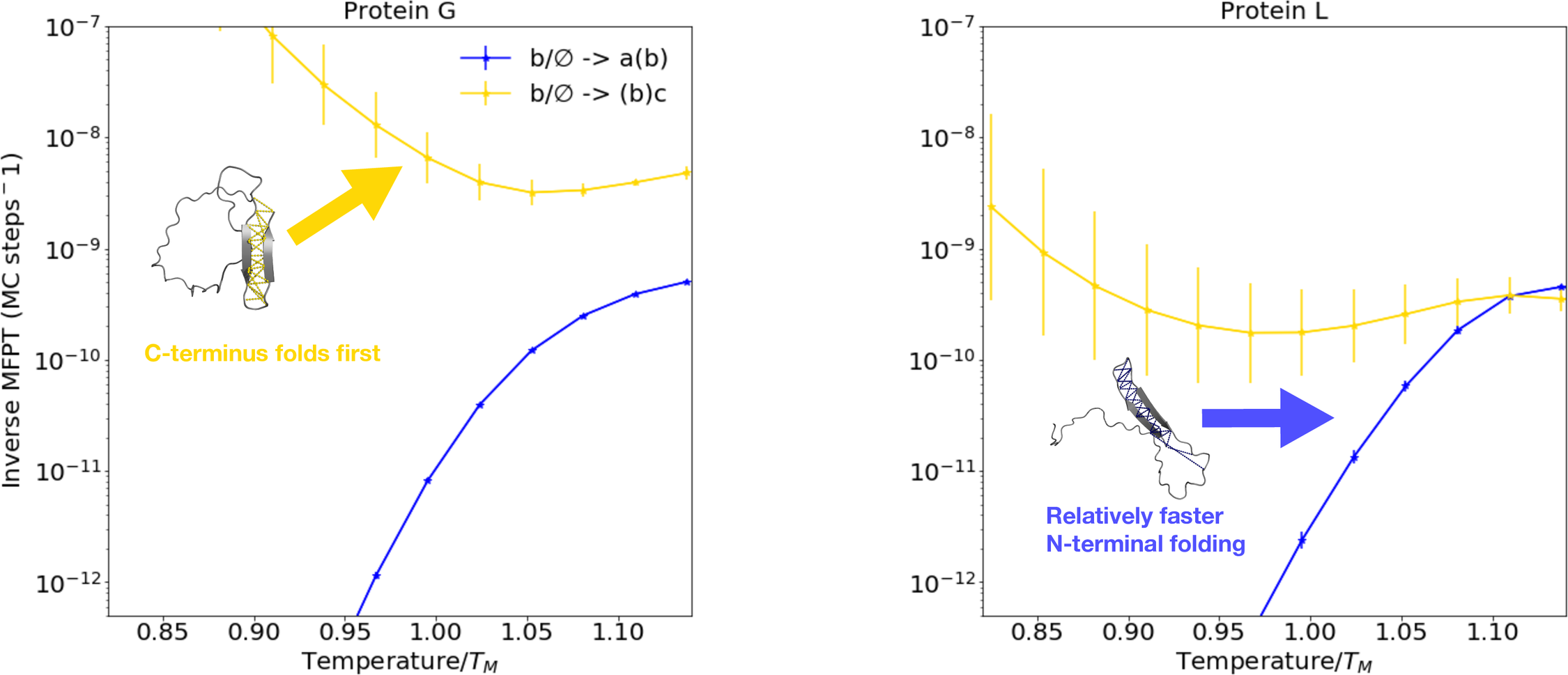

Supplement: S11 Fig — We compute potentials of mean force (PMFs) as a function of topolgoical configuration (as in main text Fig 3C) for protein G (left panels) and its close structural homolog protein L (right panels) at temperatures of T = 0.88 TM (top row) and T = 0.94 TM (bottom row), where TM is the melting temperature for protein G. Equilibrium simulations for protein L were run for ∼1.5 billion MC steps, then analyzed in an analogous fashion as for protein G (see Methods in main text), starting from the crystal structure with PDB ID 2ptl. Substructures for protein L are defined nearly identically as for protein G. As shown here, at temperatures below the melting temperature, topological configurations in which the N-terminal hairpin (substructure a) is folded but not the C-terminal hairpin (namely configurations a, ab, and abd) are lower in free energy in protein L than they are in protein G. This is consistent with phi-value analysis, which suggests that the N-terminal hairpin in isolation is more stable in the former protein. (TIF) [file pcbi.1008323.s012.tif]

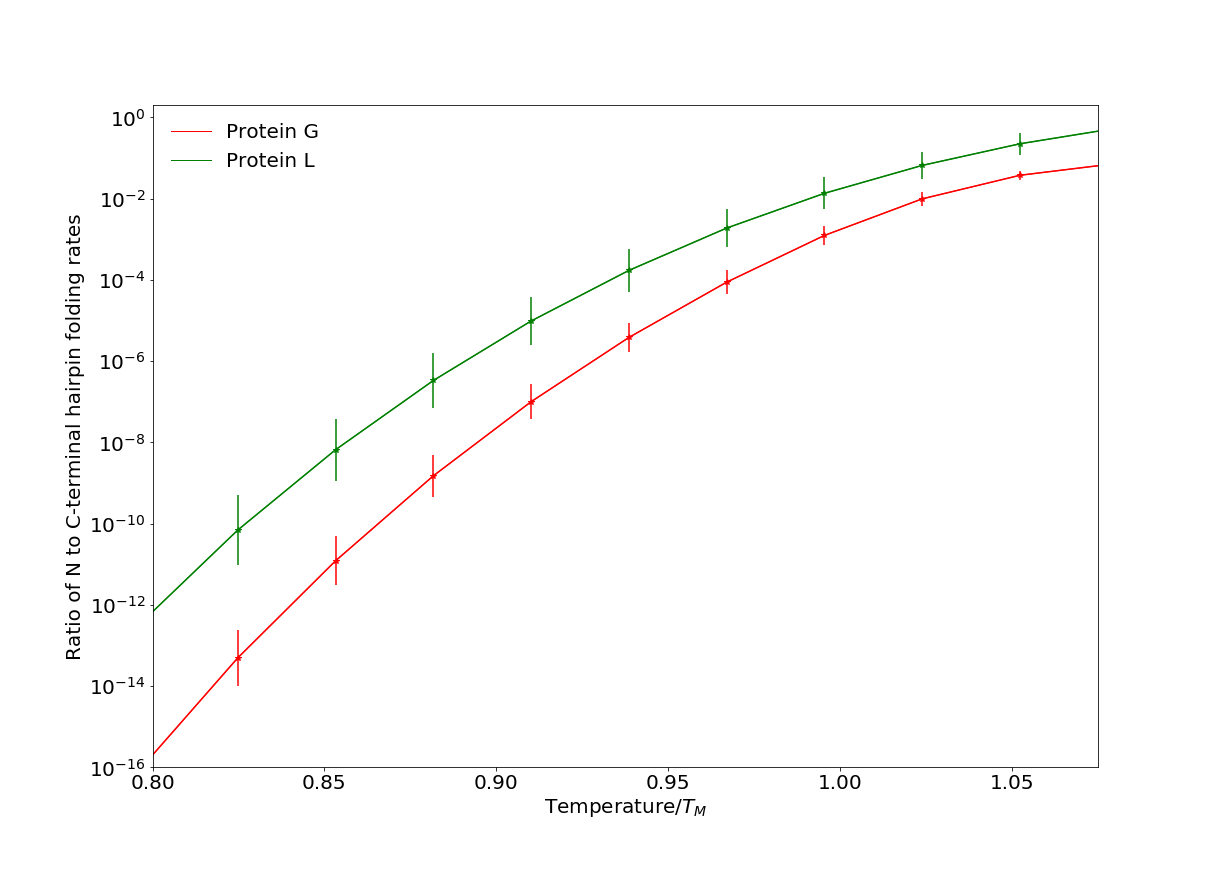

Supplement: S12 Fig — For protein G (red curve with markers and error bars) and protein L (green curve with markers and error bars), we compute the ratio of the rate at which the N-terminal hairpin folds starting from the unfolded state (transition ∅/b − >a(b)) to the rate at which the C-terminal hairpin folds (transition ∅/b − >(b)c). Error bars are obtained via bootstrapping is in main text Fig 6. We observe that for both proteins, the N-terminal hairpin’s folding is significantly slower than that of the C-terminal hairpin at temperatures below the melting temperature TM. But for protein L, this ratio is higher, indicating increased N-terminal folding as compared to protein G. Thus, although our MCPU potential does not predict a complete change in folding flux towards the N-terminal pathway in protein L, it nevertheless captures a partial shift which is potentially consistent with experimental ϕ-values. We note that for protein G, it was necessary to initialize simulations from the a(b) cluster in order to compute the N-terminal folding rate. This is because, during simulations initialized from the native state, a very low amount of flux through the N-terminal unfolding pathway was observed, thus precluding the collection of sufficient statistics for Arrhenius fitting. (TIF) [file pcbi.1008323.s013.tif]
