# Supplementary material for: Validation of DBFOLD: An efficient algorithm for computing folding pathways of complex proteins
Source: PLoS Comput Biol. 2020 Nov 16;16(11):e1008323. doi: 10.1371/journal.pcbi.1008323 (PMC7704049; doi:10.1371/journal.pcbi.1008323)
Supplement: Attachment — Submitted filename: Reviewers response.docx [file pcbi.1008323.s014.docx]

**Reviewer #1: Review

In their manuscript “DBFOLD: An efficient algorithm for computing folding pathways and rates of complex proteins”, Bitran et al present an algorithm that can “predict folding pathways and rates for a wide range of proteins while accounting for the effects of non-native contacts.”

The model underlying the algorithm is very well-described, and much care is taken to justify its assumptions (e.g. the network structure of intermediates, and how that impacts their ability to apply a detailed balance constraint to calculate mean first passage times; justification of Arrhenius kinetics).  Such care is necessary because unlike many models of folding which coarse-grain the folding landscape into contact-based states, DBFOLD considers non-native contacts as well.  

While the manuscript is a valuable description of what DBFOLD is, and the general framework of what it seeks to accomplish, the applications presented are somewhat disappointing:

(1) The abstract promises “a wide range of proteins”, but folding rates and pathways are only presented for Protein G.   Because the authors used an all-atom Monte Carlo method (MCPU) to sample the folding dynamics, rate estimates are all in units of Monte Carlo steps, which do not connect directly to experiment.  Much work has been done to validate rate predictions of molecular dynamics simulations against experimental rates and pathways – have similar benchmarks been performed for MCPU?  How can the authors say that their method is predictive of rates if not?     The authors’ comparison of predicted rates against long MCPU trajectories shows their algorithm is working correctly, not that it is predictive of experiment.**

Thank you for these useful suggestions. To highlight how our method has been applied to a wide range of proteins, we have added figure 7 and the accompanying text in the results section. Furthermore, we agree that at the our first draft failed to clarify that these MC simulations cannot predict rates in real units. To address these points regarding experimentally-testable predictions, we have added a subsection at the end of the discussion. where we discuss ways in which the relative (rather than absolute) rates predicted by MC simulations may nonetheless be used to generate predictions. We also mention that obtaining absolute rates would require calibrating the MC simulations against previously studied proteins, or modifying DBFOLD to work with molecular dynamics simulations:

When DBFOLD is used alongside Monte-Carlo simulation algorithms such as MCPU, it can predict relative rates of different folding transitions for a given protein, but not absolute rates in measurable units, as the predicted rates are all in Monte-Carlo units. Knowledge of these relative rates is nevertheless useful{for instance, it allows for rate-limiting folding step(s) to be identified. Likewise, kinetic models can be generated that predict the relative populations of different folding intermediates over time (e.g. Fig. 6E), even if the absolute timescale over which this evolution occurs is not known. In order to convert Monte-Carlo rates to experimentally-measurable units, it will be necessary to benchmark the MC simulations against experimentally measured folding times. Alternatively, DBFOLD could in principle be utilized with molecular dynamics (MD) simulations, which have the obvious advantage of predicting folding rates in absolute, measurable units…

In addition, we have changed the title from

DBFOLD: An efficient algorithm for computing folding pathways and rates of complex proteins

to

DBFOLD: An efficient algorithm for computing folding pathways of complex proteins

and we have modified parts of the abstract that previously discussed rates, to clarify:

Here, we present an algorithm, entitled DBFOLD, which can predict folding pathways for a wide range of proteins while accounting for the effects of non-native contacts. In addition, DBFOLD can predict the relative rates of different transitions within a protein's folding pathway…As a proof of principle, we show that our method can accurately predict folding pathways and Monte-Carlo rates for the well-characterized Streptococcal protein G….

Finally we changed the last sentence of introduction

In its current implementation, DBFOLD computes rates in Monte-Carlo (MC) units. This allows for meaningful comparison of the relative rates of different steps in a given protein's folding pathway. Additionally, MC folding rates can be meaningfully compared across truncated forms of a given protein in order to elucidate how vectorial synthesis affects co-translational folding [17].

**(2) The authors observe the expected order of folding for Protein G (the C-terminal hairpin folds first).  While this is a nice result, it is difficult to see whether it is truly predictive.  Experimental evidence for the order of folding events has come from measuring changes in protein stability and folding/unfolding kinetics due to site-directed mutations (i.e. “phi-analysis”).  Can DBFOLD predict *changes* in the folding rates in agreement with experiment?  Could DBFOLD distinguish between the orders of hairpin formation for Protein G vs. Protein L, for example?**

We agree that the first draft did not make it clear how our results generate experimentally-testable predictions. We added such predictions in the penultimate paragraph of the discussion:

The atomistic description of folding provided by DBFOLD can be used to generate novel experimentally-testable predictions, including the role of nonnative contacts. In the case of protein G, we observe that the N-terminal beta strand (residues 5-10) frequently docks with the C-terminal hairpin in a non-native, anti-parallel fashion early during folding (Fig. 4A). These nonnative contacts persist for a significant amount of time, sometimes causing the protein to enter the non-productive pathway 1 whereby both hairpins close while misaligned. This leads to an off-pathway kinetic trap. Although previous work has also identified nonnative states involving these hairpins docked in an anti-parallel orientation [38], our work provides a quantitative estimate of the effect of these nonnative contacts on folding kinetics. Namely, our finding that a significant fraction of the population adopts long-lived states in which the hairpins misalign in an antiparallel fashion (Fig. 6E) suggests these nonnative contacts should be observable by FRET. Our method can also be applied to predict how sequences changes affect a protein's folding pathway. For instance, previous work suggests that protein G's close structure homolog, protein L, folds via its N-terminal hairpin first [31, 32], in contrast to protein G whereby folding begins with the C-terminal hairpin. Consistent with this, DBFOLD predicts increased stability for intermediates along this N-terminal pathway in protein L as compared to protein G (Fig. S11). Moreover, we find that the ratio of the N-to-C terminal hairpin folding rates is higher for protein L than for protein G (Fig. S12), although the simulations do not predict a complete shift in the folding flux towards the N-terminal pathway. Nevertheless, we observe the expected trend between these two proteins, and we note that DBFOLD can readily be applied to simulations under alternative energy functions which may capture this particular mutational effect more thoroughly.

When used with our MCPU potential, we expect that DBFOLD may accurately predict sequence effects on the folding of proteins for which the potential has successfully predicted mutational changes before, including E. Coli DHFR [52] and human gamma-D-crystallin [53]. Finally, our method has been previously applied to generate atomistic-level predictions of how complex, misfolding-prone proteins may begin folding co-translationally [17]. These predictions can be readily tested by purifying and biophysically characterizing protein fragments of different lengths.

We likewise ran simulations of Protein L for comparison with protein G as you suggest—these results are included in this above paragraph and in the SI. Although our potential energy function does not predict a complete shift in the order of hairpin formation, we do find that this sequence change increases the stability of intermediates in which the N-terminal hairpin alone is folded. Moreover, we find that the ratio of the N-to-C terminal folding rates is higher in protein L than in protein G, in accordance with the expected trend. As mentioned in the discussion, DBFOLD can readily applied with a range of potential energy functions, and when used with MCPU, we expect it to accurately predict folding-related mutation effects for various proteins against which the potential has previously been verified [e.g. Tian et. al. PLOS Comp Bio 2015, 11(4) and Serebryany et. al. JBC 2016; 291(36)]

**Near the end of this manuscript, the authors only briefly discuss their results applying DBFOLD to larger, more complex proteins, referring to Reference 17.   It would be helpful for the reader to include some examples of larger proteins in the current manuscript – it is one of the key advantages of the algorithm.**

We thank you for this very useful suggestion. Indeed, the applicability to larger, more complex proteins with slow folding kinetics and nonnative traps is one of the key advantages of the algorithm. To highlight this, we have added figure 7 and an accompanying section to the text, “Application of DBFOLD to proteins with complex folding pathways”,

In the previous sections, we have verified that DBFOLD can accurately predict folding pathways and Monte-Carlo rates for protein G, a small protein for which these predictions can readily be verified via direct folding simulation. But for larger, more complex proteins, DBFOLD can compute folding pathways at a significantly reduced computational cost relative to direct simulation, which would require unreasonably long simulation times. We quantify this computational speedup for various proteins simulated here and in reference [17] by tallying the total time that was required to run equilibrium simulations to convergence, and to collect sufficient high-temperature unfolding statistics to allow for extrapolation prior to use of DBFOLD (Fig. 7, rightmost column). These times range from 2 to 5 _ 109 MC sweeps, which corresponds to wall times on the order of 10 CPU days on a 4x AMD Opteron 6376 2.3 GHz processor. For each protein, we compared this time to the the average time required for a direct simulation to undergo the rate-limiting folding step in the context of the MCPU potential and moveset (Fig. 7, second column to right). These times, which were estimated directly from the predicted rates obtained from DBFOLD, range from 10^6^ MC sweeps for the HEMK N-terminal domain (~12 hours of wall time) to ~10^17^ MC sweeps for MarR (_2 million years of wall time). We note that this ~ 11 order-of-magnitude range in predicted folding times is comparable to the experimentally measured range from sub-microseconds to thousands of seconds [40].

From these values, we see that DBFOLD significantly reduces the computational costs required to compute folding properties for the proteins MarR, FabG, and CMK, the latter two of which are larger than 200 AAs in size. In contrast, for the 159 AA protein DHFR, the cost to use DBFOLD is comparable to the direct simulation cost, whereas for the sub-100 AA HEMK N-terminal domain and protein G, DBFOLD is significantly less efficient than direct folding simulation. This is because these later two proteins' fast folding rates are readily accessible to direct simulation. Indeed in reference [17], we confirm that HEMK NTD folding events are observable within the timescale of a short unfolding/refolding trajectory, as is the case here for protein G. Interestingly, we note that the three proteins for which DBFOLD confers a substantial computational advantage are the same ones that were predicted in reference [17]to benefit from co-translational folding. Namely, these proteins were found to fold very slowly due to nonnative contacts involving C-terminal residues, which can be circumvented by commencing folding at intermediate chain lengths. Thus we observe that DBFOLD particularly facilitates these computations for proteins which undergo slow folding due to deep nonnative traps.

We have also made reference to this efficiency calculation at the beginning of the discussion:

Use of method to investigate folding of complex proteins

In addition to cross-validating DBFOLD's predictions against direct folding simulations of the fast-folding protein G, we have shown that this method significantly reduces the computational effort required to predict folding pathways and Monte-Carlo rates for larger proteins whose folding is significantly slowed by nonnative contacts.

**Reviewer #2: This work describes a method (DBFOLD) to extract folding and unfolding rates at room temperature based on replica-exchange simulations combined with umbrella sampling and high-temperature unfolding simulations.  The method is validated on a small protein with alpha-beta fold using Monte-Carlo simulations as sampling algorithm and a knowledge-based force-field to describe the protein.  Comparison to refolding simulation shows that DBFOLD is able to recapitulate folding rates with good accuracy.  The paper is generally well written and the methodology and underlying assumptions are described in detail.  For the most part, the method has been thoroughly tested and seems to work well. 

The main criticism I have of this work is that the manuscript completely omits to acknowledge and compare to previous studies on the same subject. Starting from the pioneering work of Daggett and Fersht, In the last 20 years, a number of methods have been proposed to extract kinetic quantities from high-temperature unfolding and/or enhanced sampling simulations.  While I don’t think a method identical to DBFOLD has been presented before, it share many similarities with existing methods, but the previous literature on the subject is completely ignored.  Just to give a few examples that come to my mind, methods to extract kinetics from REMD simulations have been developed by Andrec et al. PNAS 102:6801-6806 (2005), Van Der Spoel and Seibert PRL 96:238102 (2006), Buchete and Hummer Pys. Rev. E 77:030902 (2008), Stelzl and Hummer JCTC 13(8):3927 (2017); in the context of metadynamic simulations methods to extract kinetics have been proposed by Marinelli et al. Plos. Comp. Biol.
5(8):e1000452 (2009) and Tiwary and Parrinello PRL 111:230602 (2013). At the very least, previous studies should be properly acknowledged and differences between DBFOLD and previous work should be discussed.**

We certainly agree that a number of previous works have used similar techniques and that it was a oversight to neglect them in the first draft. We have substantially revised the first two paragraphs of the discussion to discuss how our method differs from these works, and others. In particular, we emphasize that the combined use of unfolding and equilibrium simulations allows us to address the following limitations from previous works: 1.) High-temperature simulations alone cannot reveal folding *rates*, and the folding intermediates at these high temperatures may not show nonnative contacts that are relevant under physiological conditions, and 2.) Although previous works have extracted kinetics from REMD simulations, these techniques are only expected to work for small proteins where transitions between intermediates are frequent. 3.) Obtaining an optimal order parameter for use in Metadynamics is very difficult for large proteins

We have developed a novel computational method, DBFOLD, that allows for the prediction of protein folding pathways and relative rates of different transitions while accounting for the effects of off-pathway non-native contacts. Rather than directly simulating folding, we show that one can use a combination of equilibrium simulations with enhanced sampling and unfolding simulations to compute folding rates using the principle of detailed balance. Our method builds on previous studies that made use of high-temperature unfolding simulations to shed light on protein folding pathways [43, 44]. However, these simulations by themselves cannot be used to obtain rates of folding, nor can they elucidate the role of nonnative contacts, which may stabilize folding intermediates at physiologically-relevant temperatures but be absent at high temperatures at which unfolding simulations are run

Our work overcomes this limitation by making use of replica-exchange simulations to model intermediate states under physiological conditions. We note, however, that our approach for simulating kinetically-complex proteins differs from previous efforts to extract kinetic information from replica-exchange simulations [45{48]. While these previous methods can accurately compute transition rates for small proteins that rapidly transition between conformers, larger proteins rarely undergo such transitions within simulation timescales in the absence of a biasing potential. Although methods have been developed to obtain rates from biased simulations such as our equilibrium simulations [49], these techniques require knowledge of a functional form for how bias affects transition rates, which may not be known for complex systems. Our method is the first, to our knowledge, which combines biased replica-exchange simulations with high-temperature unfolding simulations to overcome the difficulty in accurately computing transition rates for large proteins. Moreover, while we have chosen to bias our equilibrium simulations along one specific order parameter, namely native contacts, our method does not require us to have chosen an optimal order parameter along which slow transitions occur, as is the case with Metadynamics-based methods [50, 51]. Rather, the purpose of our biasing potential is to promote sampling of distinct minima along the folding landscape, and not necessarily the transition saddle points. The free energy barriers associated with these transitions are instead obtained via extrapolation from high-temperature unfolding simulations. Thus, we expect that biasing along alternative order parameters, such as root-mean squared deviation (RMSD) and, radius of gyration (RG), will yield similar results, and believe it would be interesting to compare their efficiencies in future work.

**Minor points:
DBFOLD is presented as an “efficient’ algorithm to compute folding rates, but a proper definition of efficiency is not clearly presented.  The algorithm requires fully sampling the folding free energy surface to convergence (which, even in the context of enhanced sampling, is a daunting task) and also running a number of unfolding simulations.  It would be informative to compare the computational cost of running the full REMD simulation to convergence as compared to just running unbiased plain folding simulations to estimate the folding rate.**

Thank you for this very helpful suggestion. We certainly agree that this comparison is extremely important to establish the efficiency of the algorithm. We have added figure 7 and the accompanying section, “Application of DBFOLD to proteins with complex folding pathways”, to address this. In brief, we find that the cost of using DBFOLD is significantly lower than that for brute-force simulation for those proteins that suffer from deep, long-lasting kinetic traps.

In the previous sections, we have verified that DBFOLD can accurately predict folding pathways and Monte-Carlo rates for protein G, a small protein for which these predictions can readily be verified via direct folding simulation. But for larger, more complex proteins, DBFOLD can compute folding pathways at a significantly reduced computational cost relative to direct simulation, which would require unreasonably long simulation times. We quantify this computational speedup for various proteins simulated here and in reference [17] by tallying the total time that was required to run equilibrium simulations to convergence, and to collect sufficient high-temperature unfolding statistics to allow for extrapolation prior to use of DBFOLD (Fig. 7, rightmost column). These times range from 2 to 5 *10^9^ MC sweeps, which corresponds to wall times on the order of 10 CPU days on a 4x AMD Opteron 6376 2.3 GHz processor. For each protein, we compared this time to the the average time required for a direct simulation to undergo the rate-limiting folding step in the context of the MCPU potential and moveset (Fig. 7, second column to right). These times, which were estimated directly from the predicted rates obtained from DBFOLD, range from 10^6^ MC sweeps for the HEMK N-terminal domain (~12 hours of wall time) to ~10^17^ MC sweeps for MarR (_2 million years of wall time). We note that this ~ 11 order-of-magnitude range in predicted folding times is comparable to the experimentally measured range from sub-microseconds to thousands of seconds [40].

From these values, we see that DBFOLD significantly reduces the computational costs required to compute folding properties for the proteins MarR, FabG, and CMK, the latter two of which are larger than 200 AAs in size. In contrast, for the 159 AA protein DHFR, the cost to use DBFOLD is comparable to the direct simulation cost, whereas for the sub-100 AA HEMK N-terminal domain and protein G, DBFOLD is significantly less efficient than direct folding simulation. This is because these later two proteins' fast folding rates are readily accessible to direct simulation. Indeed in reference [17], we confirm that HEMK NTD folding events are observable within the timescale of a short unfolding/refolding trajectory, as is the case here for protein G. Interestingly, we note that the three proteins for which DBFOLD confers a substantial computational advantage are the same ones that were predicted in reference [17]to benefit from co-translational folding. Namely, these proteins were found to fold very slowly due to nonnative contacts involving C-terminal residues, which can be circumvented by commencing folding at intermediate chain lengths. Thus we observe that DBFOLD particularly facilitates these computations for proteins which undergo slow folding due to deep nonnative traps.

We have also made reference to this efficiency calculation at the beginning of the discussion:

Use of method to investigate folding of complex proteins

In addition to cross-validating DBFOLD's predictions against direct folding simulations of the fast-folding protein G, we have shown that this method significantly reduces the computational effort required to predict folding pathways and Monte-Carlo rates for larger proteins whose folding is significantly slowed by nonnative contacts.

**One of the key feature of the approach is the calculation of folding rates.  In order to be able to compare simulation predictions to experiment, the method should be used in the context of MD simulations performed with explicit solvent.  I have some reservations about the applicability of DBFOLD to explicit solvent simulations and I would like to see some more discussion on this issue. A key feature of the method is the use of temperature REMD and umbrella sampling along the Q coordinate to get a full description for the folding free energy surface.  While Q is often a reasonable reaction coordinate, it is far from perfect, even for simple systems (see e.g. J. Chem. Theory Comput. 2017, 13, 5, 2086–2097) and its performance is expected to get worse for complex systems.  Also, due to the strong entropic component of the main free energy barrier, REMD is expected to provide (at best) about one order of magnitude performance improvement in protein folding (see e.g. Rosta and
Hummer JCP 131:165102 (2009) and Pan et al. JCTC 12:1360 (2016)), so it is unclear if this method may be applicable to vary large proteins, in particular in the context of explicit solvent MD simulations where simulation folding times are expected to mirror the experimental values that are often many order of magnitude slower than the times that can be reached by MD simulations.**

Thank you for this suggestion. We note that our method does not require an optimal order parameter, as we do not compute free energy barriers along it. Rather, the purpose of the biasing parameter in our case is simply to promote exploration of different *minima* on the landscape. We clarify this in the second paragraph of the discussion:

Moreover, while we have chosen to bias our equilibrium simulations along one specific order parameter, namely native contacts, our method does not require us to have chosen an optimal order parameter along which slow transitions occur, as is the case with Metadynamics-based methods [50, 51]. Rather, the purpose of our biasing potential is to promote sampling of distinct minima along the folding landscape, and not necessarily the transition saddle points. The free energy barriers associated with these transitions are instead obtained via extrapolation from high-temperature unfolding simulations. Thus, we expect that biasing along alternative order parameters, such as root-mean squared deviation (RMSD) and, radius of gyration (RG), will yield similar results, and believe it would be interesting to compare their efficiencies in future work.

Likewise we agree that this timescale issue makes it very difficult to apply MD simulations, even with the use of bias and exchange. We highlight this at the end of the discussion.

Alternatively, DBFOLD could in principle be utilized with molecular dynamics (MD) simulations, which have the obvious advantage of predicting folding rates in absolute, measurable units. …However, it is worth noting that the small simulation timesteps used in MD render these simulations much slower to run than MC simulations. Even with the use of replica exchange and enhanced sampling, the timescales required to achieve convergence in MD simulations may significantly exceed accessible computation times, particularly for large proteins [24, 58, 59].

Nevertheless, even in cases where MD simulations cannot be run to convergence, Monte-Carlo simulations can still be used to generate specific, experimentally-testable predictions when used with DBFOLD. We emphasize this in the penultimate paragraph of the discussion:

The atomistic description of folding provided by DBFOLD can be used to generate novel experimentally-testable predictions, including the role of nonnative contacts. In the case of protein G, we observe that the N-terminal beta strand (residues 5-10) frequently docks with the C-terminal hairpin in a non-native, anti-parallel fashion early during folding (Fig. 4A). These nonnative contacts persist for a significant amount of time, sometimes causing the protein to enter the non-productive pathway 1 whereby both hairpins close while misaligned. This leads to an off-pathway kinetic trap. Although previous work has also identified nonnative states involving these hairpins docked in an anti-parallel orientation [38], our work provides a quantitative estimate of the effect of these nonnative contacts on folding kinetics. Namely, our finding that a significant fraction of the population adopts long-lived states in which the hairpins misalign in an antiparallel fashion (Fig. 6E) suggests these nonnative contacts should be observable by FRET. Our method can also be applied to predict how sequences changes affect a protein's folding pathway. For instance, previous work suggests that protein G's close structure homolog, protein L, folds via its N-terminal hairpin first [31, 32], in contrast to protein G whereby folding begins with the C-terminal hairpin. Consistent with this, DBFOLD predicts increased stability for intermediates along this N-terminal pathway in protein L as compared to protein G (Fig. S11). Moreover, we find that the ratio of the N-to-C terminal hairpin folding rates is higher for protein L than for protein G (Fig. S12), although the simulations do not predict a complete shift in the folding flux towards the N-terminal pathway. Nevertheless, we observe the expected trend between these two proteins, and we note that DBFOLD can readily be applied to simulations under alternative energy functions which may capture this particular mutational effect more thoroughly.

When used with our MCPU potential, we expect that DBFOLD may accurately predict sequence effects on the folding of proteins for which the potential has successfully predicted mutational changes before, including E. Coli DHFR [52] and human gamma-D-crystallin [53]. Finally, our method has been previously applied to generate atomistic-level predictions of how complex, misfolding-prone proteins may begin folding co-translationally [17]. These predictions can be readily tested by purifying and biophysically characterizing protein fragments of different lengths.

**Another technical issue related to the applicability in the context of explicit solvent simulations is the approximations due to the use of the Arrhenius equation.  While this approximation seems to hold in the MonteCarlo simulations presented here, in explicit solvent MD simulations the prefactors may have a very strong temperature dependence and also there could be a stronger temperature dependence in the activation energies which could make the extrapolation from high-temperature unfolding simulation results more problematic.  I believe that some more discussion of potential problems may be helpful for the readers.**

Thank you for this very useful suggestion. To address this, we point out, at the end of the discussion, that the Arrhenius assumption needs to be tested when MD unfolding simulations are run. In case the approximation does not hold, alternative models which account for these additional temperature dependencies can be used.

Alternatively, DBFOLD could in principle be utilized with molecular dynamics (MD) simulations, which have the obvious advantage of predicting folding rates in absolute, measurable units. We expect the method to work well with MD so long as the requirements presented here are satisfied. In particular, it will be important to verify that unfolding rates can still be well-fit to the Arrhenius equation. The presence of explicit solvent may introduce a temperature-dependence to the unfolding-rate prefactors and activation energies, in which case an alternative model should be used for rate extrapolation [57].
