# Supplementary material for: Validation of DBFOLD: An efficient algorithm for computing folding pathways of complex proteins
Source: PLoS Comput Biol. 2020 Nov 16;16(11):e1008323. doi: 10.1371/journal.pcbi.1008323 (PMC7704049; doi:10.1371/journal.pcbi.1008323)
Supplement: Attachment — Submitted filename: Second rebuttal letter.docx [file pcbi.1008323.s015.docx]

We would like to thank the editors and reviewers for their constructive comments on our manuscript. Our responses are attached below:

Editorial board

**We thank the authors for their changes to the manuscript.  We note that the systems added to the resubmitted manuscript are not novel but have been published by the authors in their prior PNAS paper.  (To be completely forthright, given the advertised computational efficiency of this approach, we would have hoped for additional protein systems.)  Since this makes the paper more a validation study than a new results study, we would suggest that the title be prefixed with "Validation of" to read "Validation of DBFOLD" before the colon.**

Indeed, we have published results on the majority of these systems before using our method (with the exception of protein G), and so we agree that this should be presented as a validation work. We have made the suggested change to the title.

Reviewer 1

**In the revised manuscript the authors have done a good job revising the manuscript that has greatly improved.

I have just a comment about the discussion of protein G vs Protein L folding pathway, which I found a bit too qualitative. The phi-values offer a rather indirect structural information of folding pathways and transition states, and it is possible that the simulation results may actually be consistent with the experiment. To verify this, it would be necessary to compare to the raw data, ie.e folding rates and stabilities of mutants. There are ways to do this. The most accurate would be directly reproducing experimental rates and stabilities by simulation of the mutants, but this is computationally expensive. Alternatively, it may be possible to use approximate methods based on determination of the transition state for folding or of an approximate free energy landscape to infer the effect of mutations on folding rates and stabilities. I am not arguing that the authors must do this at this stage, but it may be worth at least briefly mentioning this point.**

Thank you for your supportive comments. We agree that our results may not be in contradiction to phi-value analysis, which do not always accurately predict the order of folding events, and that further computation would be necessary to allow for qualitative comparison with these experimental values. To reflect this, we have changed the relevant section in the discussion to the following:

For instance, previous work suggests that in protein G's close structure homolog, protein L, residues in the N-terminal hairpin show the highest phi-values (Kim2000). This is in contrast to protein G, where the highest phi-values are found in the C-terminal residues (McCallister2000). Potentially consistent with this, we find that DBFOLD predicts folding intermediates involving the N-terminal hairpin to be ~5-10 kBT more stable in protein L than in protein G at physiological temperatures (Fig. S11). But a more quantitative comparison with experimental phi-values would require computation of folding stabilities and transition state barriers for all the relevant mutants within our potential, either using our method or less costly, approximate methods (Jacobs2016). Our current simulations also predict that the ratio of the N-to-C terminal hairpin folding rates is higher for protein L than for protein G (Fig. S12), although the simulations do not predict a complete shift in the folding flux towards the N-terminal pathway. But this incomplete shift does not necessarily contradict experimental phi-values, which sometimes fail to unambiguously predict the structure of the transition state ensemble. For instance, studies using psi-value analysis paint a different picture whereby all four beta strands are formed in the transition states for both homologs (Baxa2015).
